# The effectiveness of a motivational enhancement smartphone application promoting lifestyle improvement for brain health: A randomized controlled trial

**DOI:** 10.1371/journal.pone.0267806

**Published:** 2022-06-30

**Authors:** Hyun Woong Roh, Hankyel Ryu, Sooin Jeong, Jieun Han, Bumhee Park, So Young Moon, Seong Hey Choi, Sang Joon Son, Chang Hyung Hong

**Affiliations:** 1 Department of Psychiatry, Ajou University School of Medicine, Suwon, Republic of Korea; 2 Department of Medicine, Ajou University School of Medicine, Suwon, Republic of Korea; 3 Department of Psychiatry, Samsung Medical Center, Sungkyunkwan University School of Medicine, Seoul, Republic of Korea; 4 Department of Biomedical Informatics, Ajou University School of Medicine, Suwon, Republic of Korea; 5 Department of Neurology, Ajou University School of Medicine, Suwon, Republic of Korea; 6 Department of Neurology, Inha University School of Medicine, Incheon, Republic of Korea; UCSI University, MALAYSIA

## Abstract

Multidomain lifestyle modification is considered an effective intervention for dementia prevention due to its multifactorial nature. Recognizing that participants’ activity adherence is crucial for successful lifestyle modification, our team developed a smartphone application to enhance motivation toward brain health behavior based on gamification theory, which influences behaviors by enhancing motivation. The developed smartphone application has two main functions: delivering supporting videos from family, friends, and medical staff, and self-evaluation. We assessed the effectiveness of this smartphone application with regard to brain health behavior. In this eight-week randomized controlled trial, 40 participants were randomly assigned to the smartphone application intervention group or control group. The primary outcome reflected participants’ brain health behavior in three categories: physical activity, cognitive activity, and healthy diet. Each brain health behavior was measured by the Korean version of the Global Physical Activity Questionnaire, Cognitive Activity Score, and Mediterranean DASH Intervention for Neurodegenerative Delay Diet Score. Furthermore, we investigated the change in motivation, measured by the Situational Motivation Scale. Additionally, we reviewed participants’ self-record diary during the first, fourth, and eighth week of intervention for evaluation of adherence. The intervention group was found to have a positive association with moderate metabolic equivalent activities (*P* = 0.01) and intrinsic motivation change (*P* = 0.01). There was a significant difference between the intervention and control groups regarding average physical activity at week 8 (*P* = 0.037). An eight-week intervention with the smartphone application induced physical activity of moderate intensity through intrinsic motivation enhancement. We suggest that the motivation enhancement application could be an efficient option for maintaining and promoting psychosocial health behavior. This smartphone application can be applied to any other disease that needs behavioral change. Through the application, a broader spectrum of the population, regardless of time, space, and human resources, can benefit from community health services.

**Trial registration:** Korean National Clinical Trial Registry CRIS identifier: KCT0005231.

## Introduction

Globally, approximately 50 million people are living with dementia [1]. This population is projected to increase to 74.7 million by 2030, reaching 131.5 million by 2050 [2]. To reduce the negative consequences of dementia for patients as well as caregivers, numerous studies have explored dementia prevention. Recent research shows that proper medication and psychological intervention can decrease the prevalence of cognitive impairment and delay its progression [3].

Multidomain interventions, owing to their multifactorial nature, are believed to be particularly effective in the context of dementia [4]. The first large-scale multidomain intervention study was The Finnish Geriatric Intervention Study to Prevent Cognitive Impairment and Disability (FINGER) trial. This randomized controlled trial clarified the effectiveness of multidomain lifestyle modification [5, 6]. Thereafter, the Worldwide FINGERS network, which adapts and optimizes the FINGER model to the region in question, spread globally [7]. Park et al.’s South Korean study to prevent cognitive impairment and protect brain health through lifestyle intervention in at-risk older adults (SUPERBRAIN) is also part of the Worldwide FINGERS network [8].

However, as the FINGER trial has shown, participants’ adherence to lifestyle modification programs can be highly variable [9]. Generally, participants are likely to discontinue dementia prevention programs owing to the relatively long duration. Adherence to lifestyle modification has been considered crucial for successful intervention [10]. We suggest that additional intervention regarding adherence may be a valuable strategy for successful dementia prevention [6, 9].

Gamification theory is a method that applies gaming mechanisms to non-gaming contexts. Gamification is known as an effective tool for influencing behavior via the enhancement of motivation, especially intrinsic motivation [11, 12]. Traditionally, gamification has been used in the fields of business, marketing, and education; however, recently, it has also been applied to healthcare [12]. Studies show that gamification has a positive impact on health-related interventions. A systematic review concluded that evidence is strongest for the use of gamification to achieve targeted behavioral outcomes, particularly physical activity [11].

It has been found that community mental health services have few beneficiaries owing to limited economic and human resources [10, 13]. Therefore, we believe that using a digital tool to deliver such services can allow a broader spectrum of the population to access them, regardless of time, space, and human resources. Owing to smartphones’ accessibility, several attempts have been made to promote health behavior through smartphone applications [14]. A meta-analysis even found that behavioral change techniques can be more effective when combined with digital tools [15]. Considering the abovementioned facts, our team developed a smartphone application to enhance motivation toward brain health behavior based on gamification.

The main aim of the present study was to test the effectiveness of an eight-week smartphone application-based motivation enhancement program for inducing brain health behavior. Secondarily, we investigated the change in motivation to engage in brain health activities.

## Materials and methods

### Recruitment

**Fig 1:**
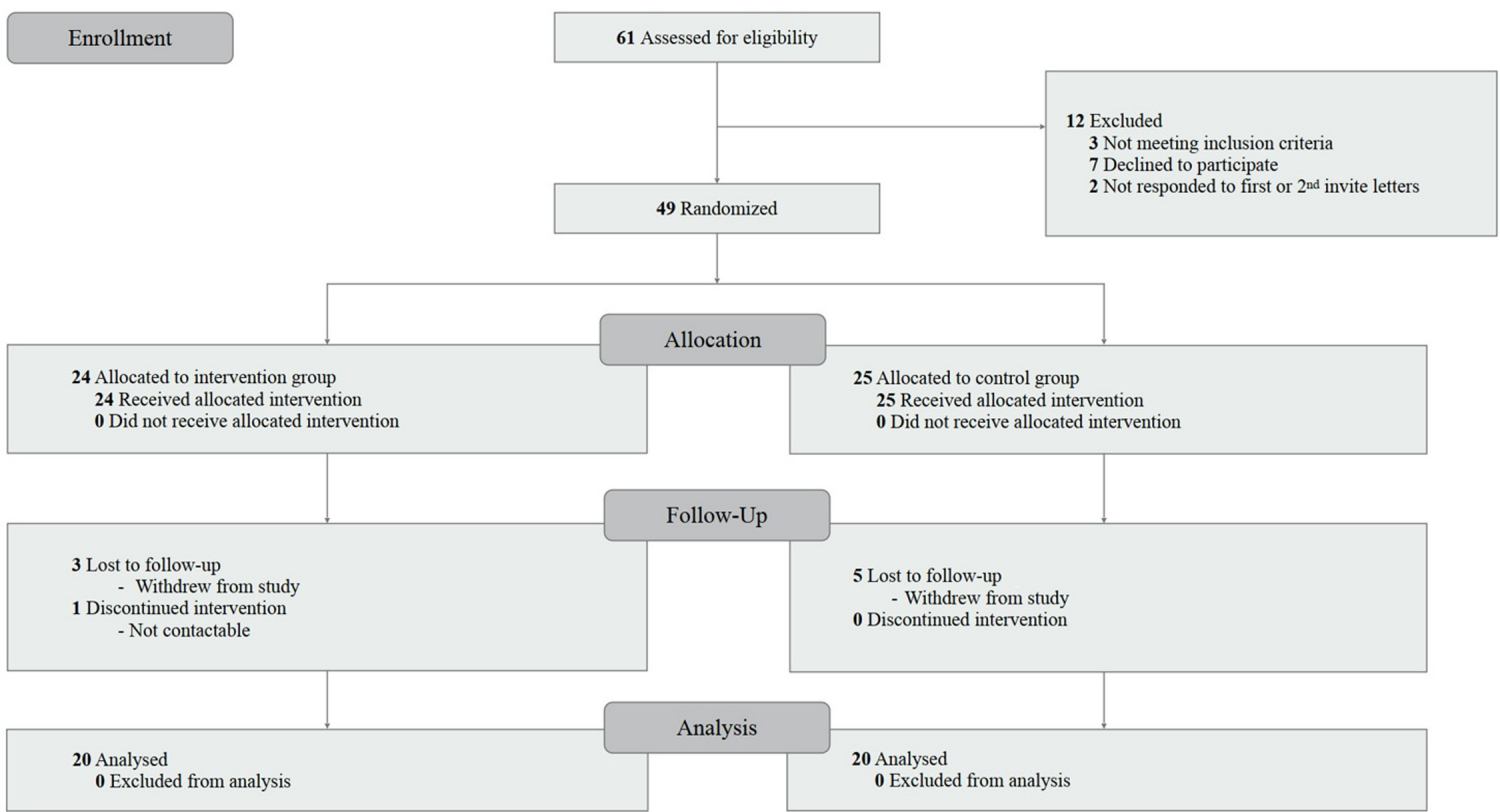
Flow chart of the study.

The current study was conducted at Ajou University Hospital, Republic of Korea. A total of 61 participants from the tertiary hospital’s outpatient clinic were assessed. Three patients were excluded after eligibility assessment, seven declined to participate, and two did not respond to letters. Therefore, after excluding these 12 patients, a total of 49 participants were randomly assigned either to the experimental group (n = 24) or the control group (n = 25) by block randomization (Fig 1).

Inclusion and exclusion criteria were as following. Inclusion criteria were: (1) aged 60 years and older; (2) users of an Android phone with an internet connection; (3) person without dementia; (4) having reading and writing ability that can be identified by a literacy test; and (5) having a reliable informant who can provide investigators with requested information. Exclusion criteria were: having [1] a history of a psychiatric disorder (mental retardation, schizophrenia, and bipolar disorder); (2) a diagnosis of dementia by the study physician; (3) a history of other neurodegenerative diseases including Parkinson’s disease; (4) a serious medical condition that can disturb study completion, (e.g. not fully recovered from a malignant cancer in five years, underwent revascularization or stent placement in a year, severe or unstable cardiovascular diseases, and other severe or unstable medical conditions including acute and severe asthma, active gastric ulcer, severe liver disease, and severe kidney disease that needs hemodialysis); (5) any conditions preventing cooperation as judged by the study physician; or (6) any conditions that could interfere with the study.

### Standard protocol approvals, registrations, and patient consents

This study is registered at the Korean National Clinical Trial Registry CRIS (identifier: KCT0005231). The study was approved by the Ajou University Institutional Review Board (AJIRB-MED-SUR-20-175). All participants and their informants signed written informed consent.

### Study design

An eight-week randomized controlled parallel-design study was conducted. For participants who met the above-mentioned inclusion and exclusion criteria, a baseline evaluation was made. All the participants were allocated to either the intervention group with the motivation enhancement smartphone application or the control group. Block randomization was done using SAS (PLAN procedure). Participants were asked to visit after the eight-week program, within two weeks from the completion. Participants could get examinations for study and brain health behavior education without charge.

### Motivation enhancement smartphone application

The smartphone application was developed and designed to enhance motivation of participants based on gamification theory. The smartphone application comprised two main functions. First, the participants could receive videos offering support from “Family-coach” through the application. Family-coach are participants’ family, friends, medical staffs, case manager, and anyone surrounding the participants who participate in reinforcing the motivation. Each Family-coach only needs to invest 10 minutes in making a video, which encouraged positive brain health behavior among the participants. Family-coach could easily upload their video through the smartphone application. Participants received the video once a week and could watch it repeatedly. Furthermore, the smartphone application sends a push alarm to watch video to remind participants. Second, the participants could evaluate their effort on brain health behavior and track their weekly self-rated achievement through the application. In our last community based randomized trial, we applied a symbolic medal system to a multidomain intervention of major depressive disorders [10]. We applied symbolic medal system to the smartphone application. Achievement levels were categorized as gold and silver medals; participants selected either one during self-rating. Participants could see their achievement in the leader board of the smartphone application. The leader board can stimulate participants to achieve a goal as last week, or to make an improvement than last week.

### Intervention

Participants of both control group and intervention group got an 80-minutes education session on brain health behavior. They learned about basic knowledge of dementia including definition, symptoms, and severity. Participants watched an education video highlighting the necessity of brain health behavior. The education video was developed based on learning and nudge theories [8].

Moreover, all participants received a manual on brain health activity regarding three main categories: physical activity, cognitive activity, and healthy diet. It also described the procedure of physical activities in detail. Physical activities were divided into three levels: high, moderate, and low intensity. High-intensity activities included hiking and aerobics; moderate-intensity activities included swimming and fast walking; low-intensity activities included slow walking and finger plays. Cognitive activity included activities like reading books, newspapers, and magazines; writing a letter, diary, and postcard; playing a puzzle, card game, and board games; and learning computers and foreign languages, drawing, and gardening. Following healthy diet indicates having five ingredients in their meal: vegetables, fish, nuts, fruits, and dishes cooked with olive oil. Participants were asked to keep a diary noting any instances where they completed each category of brain health activities. The intervention group utilized the smartphone application in addition to the education and brain health behavior activity manual.

### Outcome measurements

An intervention-induced brain health behavior was the primary outcome of interest. We measured each category of behavior, such as physical, cognitive, and healthy diet. Physical behavior change was measured using the Korean version of the Global Physical Activity Questionnaire (K-GPAQ) [16]. Cognitive behavior change was measured using the Cognitive Activity Score [17]. Healthy diet behavior change was measured using the Mediterranean DASH Intervention for Neurodegenerative Delay Diet Score (MIND Diet score) [18]. Furthermore, intervention-induced motivation change was measured using the Situational Motivation Scale (SIMS) [19]. In addition to investigating participants’ adherence, we also reviewed their self-record diary at one, four and eight weeks since the program.

### Measurement of other variables

At baseline assessment, the Korean-Mini Mental State Examination (K-MMSE) [20] was used to evaluate cognitive function. Korean version of the Geriatric Depression Scale (SGDS-K) was also conducted [21]. Big Five Inventory-Korean version-10 (BFI-K-10) and temporal discounting were conducted for adjusting participants’ personalities [22, 23]. To assess readiness of participants, the Readiness to Change Questionnaire and Theory of Planned Behavior Questionnaire were used [24, 25]. Participants completed the same tests for baseline assessment at the end of the study. Additionally, they completed Behavioral intention for brain health questionnaire. It contains seven scale questions asking participants’ perception on dementia prevention activities. There was a questionnaire about satisfaction on eight-week program.

### Statistical analysis

We used descriptive statistics in this study. Continuous variable data were reported as the mean with the standard deviation (SD) or median with the interquartile range (IQR) after verifying a normal distribution using the Shapiro-Wilk test. For group comparisons, we used the Student’s t-test and the Mann-Whitney U test for variables exhibiting a normal or non-normal distribution. For categorical variables, we examined group differences using the chi-squared test and for exploratory analyses, we performed Pearson’s correlation tests. We performed multiple linear regression analyses to examine the significance of observed association. To adjust possible covariates with parsimonious models, we used forward stepwise methods. The criterion for covariate selection involved including variables p < 0.05 and excluding variables p > 0.10. Statistical analysis was performed using SPSS software (SPSS Inc., Chicago, IL, USA).

## Results

### Baseline characteristics of participants

A flow chart of study participants is presented in Fig 1. A total of 61 participants were assessed for eligibility, and 12 were excluded. The remaining 49 participants were randomly classed into the control group (*n* = 25) or intervention group (*n* = 24). In the control group, five participants were lost to follow up. In the intervention group, three participants were lost to follow up and one participant discontinued with a giving reason. Finally, 20 participants of each group were analyzed. Participant demographic and clinical characteristics are shown in Table 1.

**Table 1:** Demographic characteristics of participants.

		Categories based on group		
Characteristics	All participants (n = 40)	Control group (n = 20)	Experimental group (n = 20)	*P* value a
**Age, mean (SD), year**	73.3 (66.8–79.8)	76.9 (72.1–81.7)	69.7 (63.7–75.7)	< .001
**Female (%)**	26 (65.0)	13 (65.0)	13 (65.0)	1.000
**Education, median (IQR), year**	10.0 (6.3–14.8)	9.5 (6.0–12.0)	11.0 (9.0–15.8)	0.201
**MMSE, median (IQR)**	27.0 (26.0–28.8)	27.0 (25.0–28.8)	27.5 (26.3–28.8)	0.429
**GDS, median (IQR)**	2.0 (1.0–4.5)	2.0 (1.0–4.5)	2.0 (1.0–4.5)	0.698
**Family history of dementia (%)**	9 (22.5)	7 (35.0)	2 (10.0)	0.127
**Comorbidity (%)**				
Diabetes mellitus	6 (30.0)	2 (10.0)	4 (20.0)	0.661
Hypertension	20 (50.0)	12.0 (60.0)	8.0 (40.0)	0.206
Dyslipidemia	9 (22.5)	6 (30.0)	3 (15.0)	0.451
Cardiovascular disease	3 (7.5)	1 (5.0)	2 (10.0)	1.000
Thyroid disease	5 (12.5)	3 (15.0)	2 (10.0)	1.000

MMSE = Mini Mental Status Examination; GDS = Geriatric Depression Scale.

a Student’s t-test was performed for normally distributed continuous variables, and the Mann-Whitney U test was conducted for continuous variables that did not exhibit a normal distribution. Chi-square tests were performed for categorical variables.

### Effects of motivation enhancement smartphone application on brain health behavior

We investigated changes of three categories of brain health behavior: physical activity, cognitive activity, and healthy diet. To investigate physical brain health activity, we assessed changes in the K-GPAQ score. The intervention group was found to have a positive association with moderate MET activities after adjusting for age, sex, education years, K-MMSE score, SGDS-K score, and BFI-K-10 score (*P* = 0.01, Table 2). There were no significant associations in cognitive activity and following healthy diet between the two groups.

**Table 2:** Multiple linear regression analysis for associations of motivational enhancement smartphone application with behavioral changes on brain health activities.

	Standardized coefficient		*P* value a
Dependent variables	Standardized β	SE	
**Physical activity change (GPAQ)**			
Moderate MET	0.44	0.15	0.01
Vigorous MET	-0.15	0.16	0.35
**Cognitive activity change (Cognitive Activity Score)**	0.26	0.16	0.11
**Dietary activity change (MIND Diet Score)**	0.14	0.15	0.34

GPAQ = Global Physical Activity Questionnaire; MET = Metabolic Equivalent; MIND diet = Mediterranean DASH Intervention for Neurodegenerative Delay diet

^a^ *P* values were adjusted for age, sex, education years, Mini Mental Status Examination score, Geriatric Depression Scale, Korean version of Big Five Inventory using a forward stepwise method.

### The effects of motivation enhancement smartphone on motivation changes

To evaluate motivation change, we assessed SIMS in each group. The intervention group was found to have a positive association with intrinsic motivation change after adjusting for age, sex, education years, K-MMSE score, SGDS-K score, and BFI-K-10 score (*P* = 0.01, Table 3). There were no significant associations with amotivation, external regulation, and identified regulation change.

**Table 3:** Multiple linear regression analysis for associations of motivational enhancement smartphone application with motivation changes.

	Standardized coefficient		*P* value a
Dependent variables	Standardized β	SE	
**Motivation change (SIMS)**			
Intrinsic motivation	0.44	0.17	0.01
Identified regulation	-0.21	0.16	0.20
External regulation	0.17	0.16	0.28
Amotivation	0.09	0.16	0.60

SIMS = Situational Motivation Scale.

^a^ *P* values were adjusted for age, sex, education years, Mini Mental Status Examination score, Geriatric Depression Scale, Korean version of Big Five Inventory using a forward stepwise method.

### Effects of motivation enhancement smartphone application on adherence

**Fig 2:**
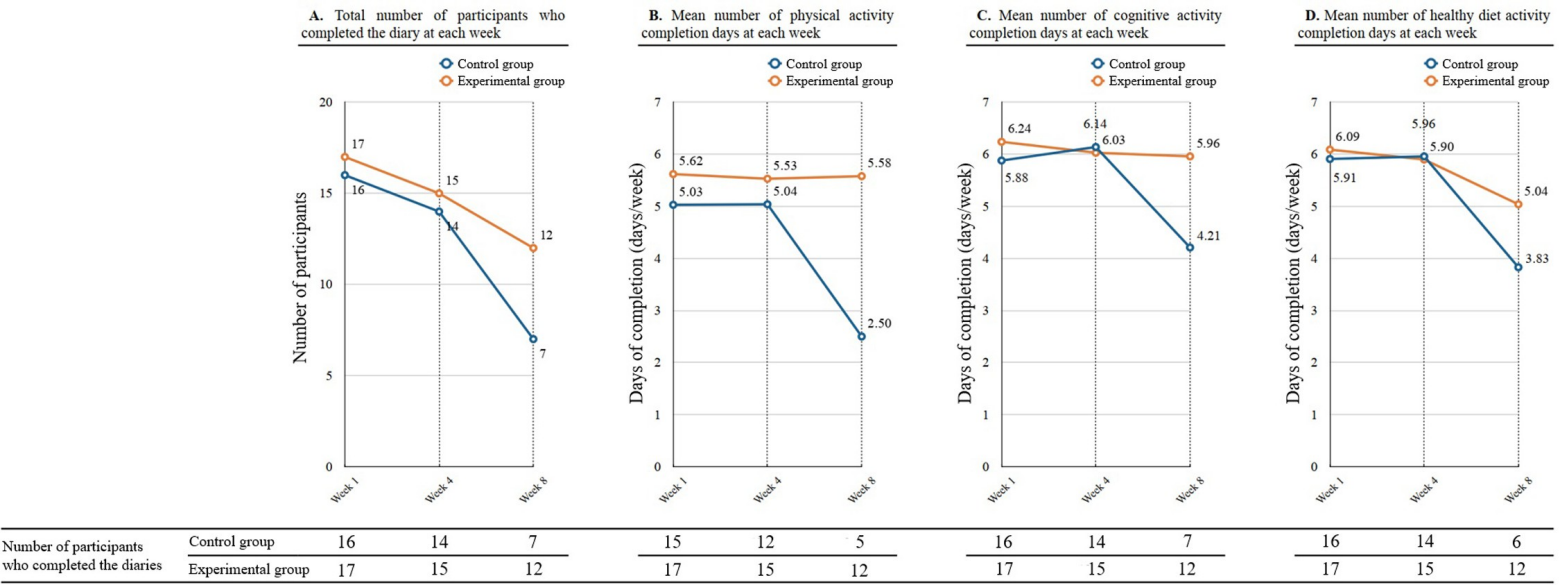
Activity completion recorded on daily diaries: Physical, cognitive, and healthy diet.

We reviewed participants’ self-record diary for investigating their adherence to the intervention program. We calculated mean number of activities completed based on the diary at first, fourth and eighth week (Fig 2). Total number of participants who checked the diary dropped sharply in the control group each week (Fig 2A). In all three categories, the mean completion days of the intervention group was more maintained than in the control group. A significant difference regarding the average physical activity completed was observed in week 8 (Fig 2B, Mann-Whitney U test, *P* = 0.037). However, there was no statistically significant difference on the cognitive activity or healthy diet activity (Fig 2C and 2D).

## Discussion

In this study, we assessed the effectiveness of the motivation enhancement smartphone application on brain health behavior. We found that there is a positive association between the smartphone application intervention and physical brain health activity of moderate intensity. Moreover, the intervention group was found to have a positive association with intrinsic motivation. Furthermore, there was a significant difference of self-reported physical activity completion between the intervention and control groups at the eight-week mark.

It is known that lifestyle modification is an effective way to prevent dementia [4]. Several lifestyle modification trials were conducted including FINGER [5], SUPERBRIAN [8], the Gold Medal Program [10], and Community Based Multidomain Lifestyle Modification on Cognitive Function [26]. However, we found that adherence was a big challenge in the face of lifestyle modification [10]. Many studies have been conducted on motivation enhancement for decades, such as Health belief model [27], self-efficacy theory [28], gamification theory [11], and self-determination theory [29].

As described above, using the smartphone application seems to be associated with moderate-intensity physical activity. To increase physical activity through the smartphone application, we suggest enhanced intrinsic motivation of the intervention group as a possible reason for why participants performed additional moderate-intensity physical activity. Intrinsic motivation is defined as “to seek out novelty and challenges, to extend and exercise one’s capacity, to explore, and to learn” [29]. An individual’s interest, enjoyment, and internal satisfaction are crucial factors to engage in a particular behavior [30]. These factors are contained in our motivation enhancement smartphone application. The smartphone application is essentially developed based on gamification theory. Each week is symbolized as one round of game, and participants completed each round by doing brain health activities. Gamification can increase participants’ interest and enjoyment, thereby encouraging positive brain health behavior.

Internal satisfaction is considered an important factor of internal motivation. Specifically, three psychological needs—autonomy, relatedness, and competence—can play pivotal roles in enhancing intrinsic motivation [29]. Participants can satisfy three psychological needs above through the smartphone application developed in this study. First, participants autonomically set a goal from the provided manual and can choose activities accordingly. Participants can self-evaluate using a symbolized medal system. Second, the smartphone application contains supporting videos that can satisfy the participants’ psychological need of relatedness. Through the supporting videos, they can emotionally connect with others. The participants can recognize brain health management as teamwork with their Family-coach. Third, using a smartphone application itself can provide participants with competence to deal with new technology. A smartphone application can work as novelty-seeking and curiosity-inducing activity, especially for elderly participants. It can lead to self-efficacy enhancement of participants.

However, there are several limitations in our study. First, due to a relatively small sample size, participants may not have been representative of the entire population. Second, there was a difference in age between participants in the control and experimental groups. However, age was adjusted as a covariate in statistical analysis. Third, the study was an eight-week period trial, and eight weeks might be a relatively short time to express brain health behavior.

Taken together, use of the motivation enhancement smartphone application is associated with moderate-intensity physical activity. The study found an association between usage of smartphone application and intrinsic motivation. We suggest that the motivation enhancement application could be an efficient option for maintaining and promoting psychosocial health behavior. Additionally, as our smartphone application is not restricted to dementia, it can be applicable on many other diseases that need behavioral change. Further research is required to evaluate effectiveness of the smartphone application in other diseases.

## Supporting information

S1 Dataset (XLSX)
